# The Silent Presentation of Gallstone Ileus in a Surgery-Naïve Abdomen: A Case Report

**DOI:** 10.7759/cureus.91310

**Published:** 2025-08-30

**Authors:** Luis M Juárez Ochoa, Michael V Benitez

**Affiliations:** 1 General Surgery, Hospital de Especialidades No. 14, Adolfo Ruiz Cortines, Instituto Mexicano del Seguro Social (IMSS), Veracruz, MEX; 2 General Surgery, Hospital General de Zona 11 (HGZ11) Instituto Mexicano del Seguro Social (IMSS), Xalapa, MEX

**Keywords:** entero-biliary fistulae, enterolithotomy, gallstone ileus, pneumobilia, rigler's triad

## Abstract

As an uncommon etiology of bowel obstruction that predominantly affects the elderly, gallstone ileus warrants attention because it frequently arises from cholecystoenteric fistulas and carries disproportionate morbidity when recognition is delayed. This case report details an unusual presentation in an elderly male patient with no history of abdominal surgery who exhibited nonspecific abdominal symptoms. Imaging studies identified Rigler's triad, and computed tomography (CT) confirmed the presence of an ectopic gallstone in the proximal ileum. The patient promptly underwent surgical intervention, including segmental bowel resection due to transmural ischemia, followed by primary anastomosis. His postoperative recovery was uneventful. This case emphasizes the importance of early recognition of gallstone ileus, highlights the diagnostic value of CT, and demonstrates that bowel resection may be necessary when bowel compromise is present. Increased awareness among clinicians can enhance outcomes and reduce complications in similar cases.

## Introduction

Defined as mechanical obstruction from a migrated gallstone via a cholecystoenteric fistula, gallstone ileus remains rare, yet clinically relevant in the elderly [1]. The clinical presentation often includes intermittent and nonspecific symptoms, causing diagnostic delays that increase morbidity and mortality [2]. Such delays frequently arise from subtle initial radiological findings, and the symptoms overlap with those of other intestinal obstructions. Clinically differentiating gallstone ileus from other forms of bowel obstruction remains challenging, especially among elderly patients whose clinical manifestations can be subtle or nonspecific. A careful history of intermittent obstructive symptoms ("tumbling obstruction"), prior biliary symptoms, and imaging features can be instrumental in differentiating gallstone ileus from other common causes such as adhesions, neoplasms, or volvulus [3-5]. The Csendes classification describes Mirizzi syndrome in five types, with type V defined as the coexistence of cholecystoenteric fistula and gallstone ileus; type Vb specifically involves multiple gallstones and small bowel obstruction [6]. This classification system provides a useful framework for understanding the pathophysiological continuum from chronic cholecystitis to fistula formation and gallstone migration. In our patient, persistent inflammation and mechanical erosion from the impacted gallstones led to the development of a cholecystoduodenal fistula, allowing the passage of a large stone into the intestinal lumen, where it became impacted in the proximal jejunum, causing obstruction.

This case emphasizes the crucial role of timely diagnosis and surgical intervention in managing gallstone ileus, along with its pathophysiological link to Csendes type Vb Mirizzi syndrome. Although rare, this condition is of clinical importance and is characterized by the development of a cholecystoenteric fistula, which subsequently causes small bowel obstruction [6]. Surgical treatment is fundamental to address gallstone ileus. While enterolithotomy is the most commonly performed procedure, segmental bowel resection is warranted in cases where ischemia, inflammation, or stricture formation is evident [7]. Prompt identification and suitable surgical management in these scenarios can substantially enhance patient outcomes and decrease perioperative morbidity and mortality [7-11]. Furthermore, CT scanning remains the cornerstone of diagnosis, as reinforced by recent evidence demonstrating its high sensitivity and specificity for detecting Rigler’s triad in suspected gallstone ileus [12].

## Case presentation

An 83-year-old man, with no history of abdominal surgery or chronic medical conditions such as diabetes or hypertension, and no previous episodes of abdominal pain, presented with a three-day history of diffuse, non-localized abdominal pain, constipation, and a single episode of non-bilious vomiting. He denied experiencing fever or hematochezia. Upon examination, the patient appeared mildly dehydrated and had a distended yet soft abdomen with intermittent cramps. Bowel sounds were hyperactive, and there were no signs of peritoneal irritation, such as rebound tenderness or evidence of an abdominal wall hernia.

**Figure 1:**
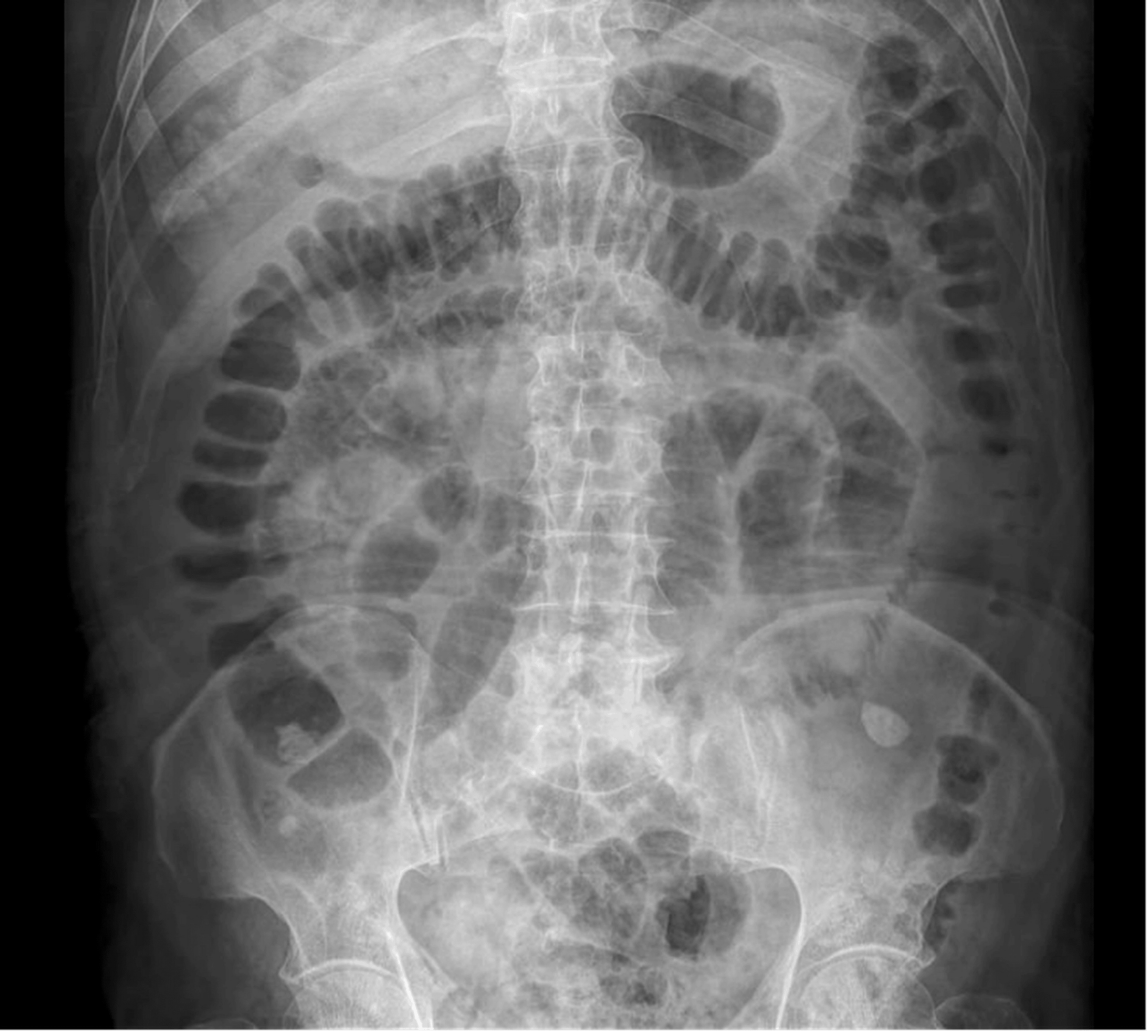
Plain abdominal X-ray demonstrating signs of small bowel obstruction An erect abdominal X-ray reveals multiple dilated loops of small intestine, air-fluid levels, and colonic gas displaced to the periphery. Subtle radiolucency in the right upper quadrant suggests pneumobilia, consistent with Rigler's triad in gallstone ileus.

Initial laboratory workup revealed leukocytosis (13.9 × 10³/μL), marked neutrophilia (12.5 × 10³/μL), and mild hyponatremia (132 mmol/L), suggestive of systemic inflammation and fluid-electrolyte imbalance. A plain abdominal radiograph demonstrated multiple dilated loops of the small bowel, with air-fluid levels and displacement of colonic gas to the periphery (Figure 1). Additionally, a radiolucent focus in the right upper quadrant indicated pneumobilia. These findings fulfilled Rigler's triad, which is classically associated with gallstone ileus.

**Figure 2:**
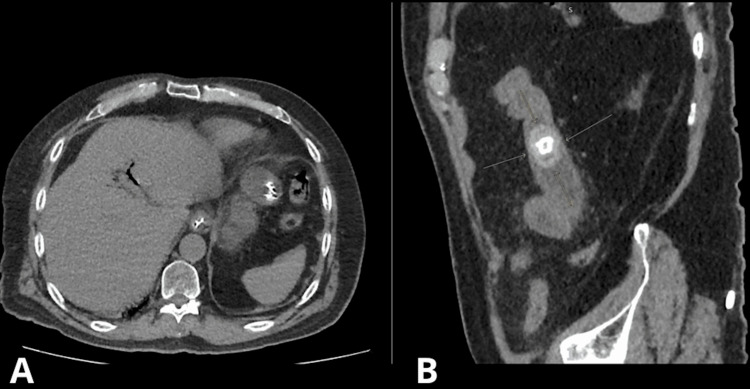
Computed tomography views demonstrating Rigler's triad in gallstone ileus (A) Axial CT image showing pneumobilia, identified as branching gas lucencies within the intrahepatic biliary tree. (B) Sagittal CT reconstruction revealing an ectopic gallstone within the small bowel lumen, exhibiting the typical radiolucent center and hyperdense rim (Rigler's triad: bowel obstruction, ectopic gallstone, and pneumobilia).

Subsequent contrast-enhanced computed tomography (CT) confirmed the diagnosis by identifying pneumobilia, dilated bowel loops, and a 30 mm ectopic gallstone located in the proximal ileum, approximately 110 cm proximal to the ileocecal valve (Figures 2A, 2B).

**Figure 3:**
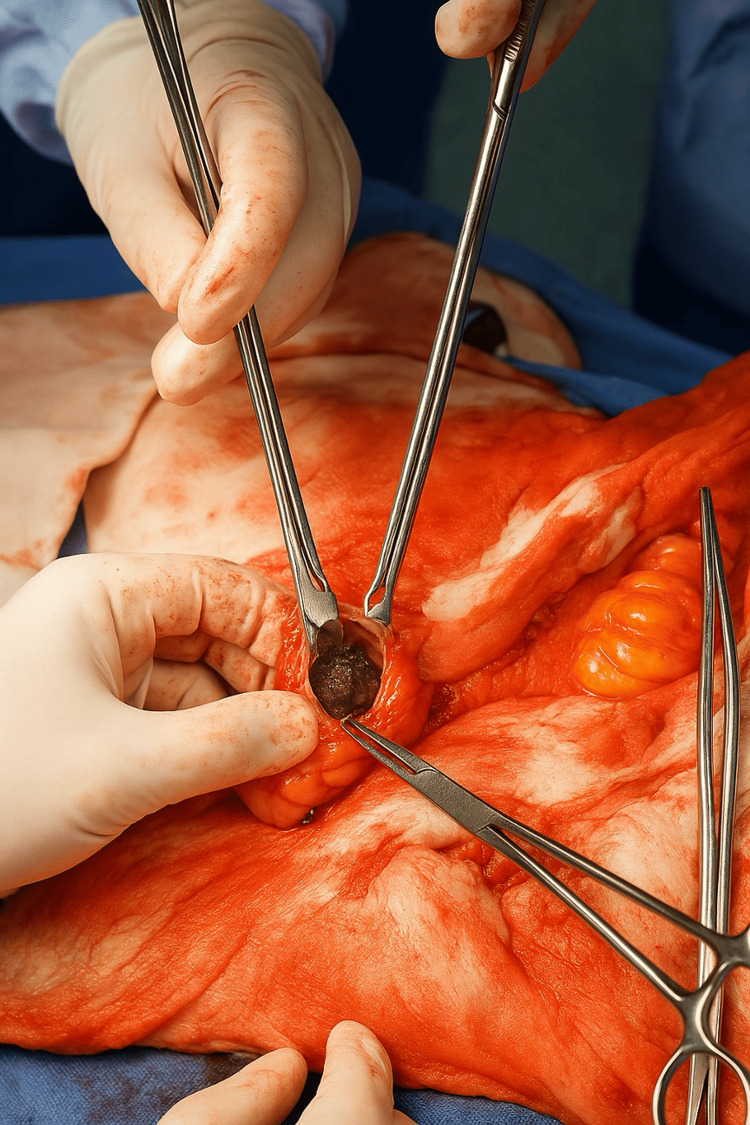
Enterotomy and removal of the obstructing gallstone Intraoperative image showing the extraction of a single obstructing gallstone through a longitudinal enterotomy, confirming the mechanical origin of the small bowel obstruction.

An exploratory laparotomy was performed after transfer. Intraoperatively, a single ectopic gallstone was found impacted in the proximal ileum, which was extracted by a longitudinal enterotomy (Figure 3).

**Figure 4:**
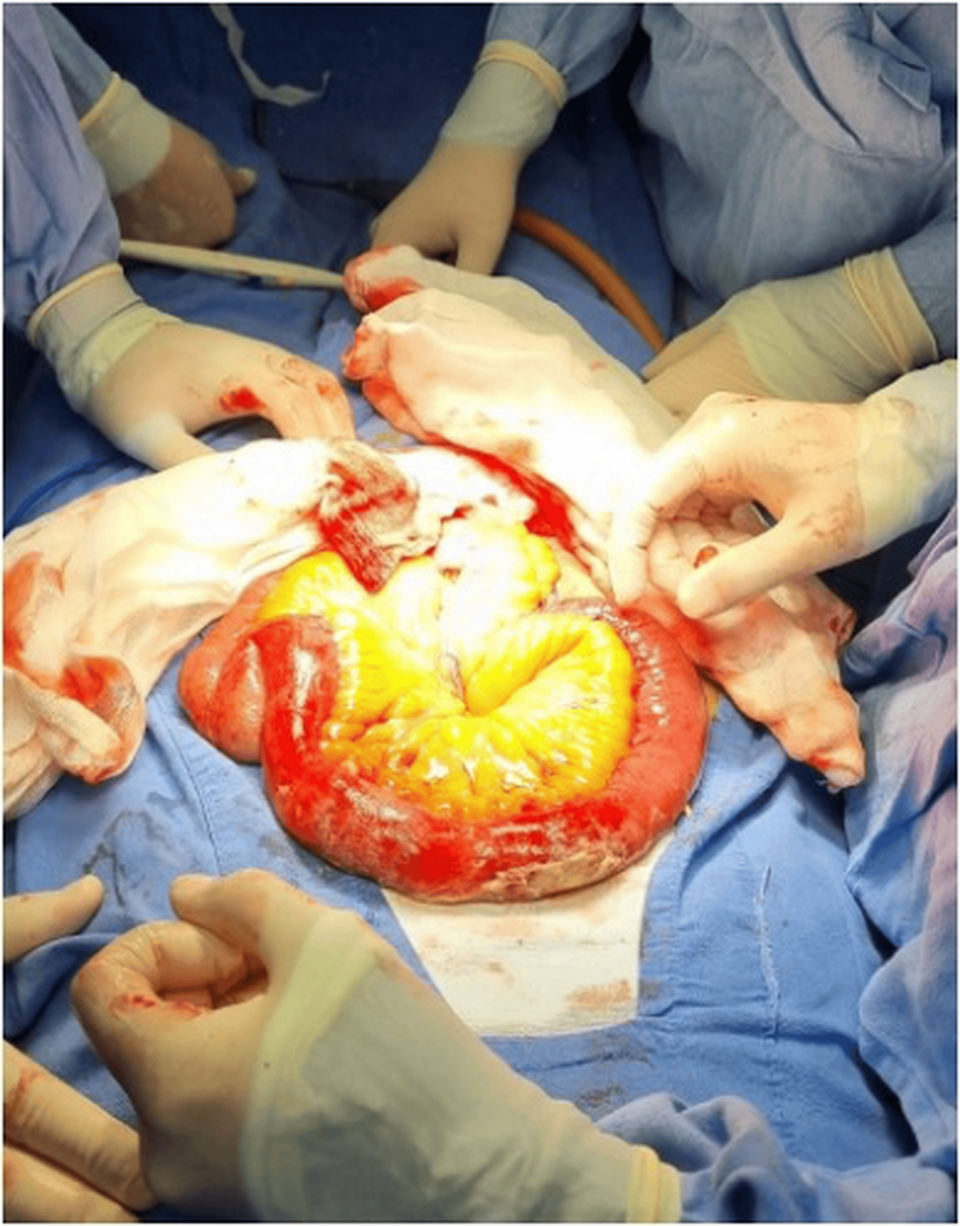
Segmental bowel ischemia requiring limited resection The affected bowel segment exhibits loss of peristalsis, congestion, and dusky discoloration, consistent with transmural ischemia. Poor viability and the potential risk of future stenosis warranted segmental resection and primary end-to-end anastomosis.

Prior to primary closure of the enterotomy, inspection revealed transmural bowel wall damage, with no visible or palpable peristaltic waves after a two-minute observation period and no response to gentle warm-compress stimulation, and early ischemic changes involving an approximately 15-cm ileal segment located approximately 120 cm proximal to the ileocecal valve (Figure 4). Given these findings, limited segmental resection of the affected bowel was performed, followed by primary end-to-end anastomosis to restore intestinal continuity. The cholecystoenteric fistula was not explored, and neither cholecystectomy nor fistula closure was undertaken, given the higher perioperative risk of a one-stage procedure in these cases.

The patient's postoperative course was uneventful. He progressed from a clear liquid to a soft diet, achieved early ambulation, and had a return of bowel function without signs of anastomotic leakage or infection. He was discharged on postoperative day five in a stable condition, with full recovery of gastrointestinal function, and no early complications were observed during outpatient follow-up.

## Discussion

Gallstone ileus is an uncommon cause of mechanical intestinal obstruction, most often described in elderly women owing to the higher prevalence of chronic cholelithiasis in this group [1]. In contrast, our patient was an elderly man, a less frequently reported demographic, providing a patient-specific reason to document this case. Compounding the rarity, the presentation was a "silent surgical abdomen," characterized by intermittently quiescent, nonspecific symptoms with few localizing signs, which directly obscured early clinical suspicion and heightened the risk of diagnostic delay.

CT is the modality of choice for suspected gallstone ileus [4,5]. In our case, CT demonstrated Rigler's triad, small bowel obstruction, pneumobilia, and an ectopic gallstone, facilitating prompt recognition and operative planning (Figures 2A, 2B) [4,6,8]. Given the patient's frailty and perioperative risk, a staged strategy was selected: enterolithotomy with limited small bowel resection without concomitant cholecystectomy and fistula repair, to safely resolve the acute obstruction and ischemia but not eliminate the possibility of recurrence or biliary complications while the cholecystoenteric fistula remains in situ [9-11]. However, our patient experienced uncomplicated postoperative recovery, underscoring the importance of rapid intervention guided by accurate CT findings. This highlights the value of timely imaging and surgical decision-making in reducing morbidity and mortality in gallstone ileus [4,7].

Surgical management of gallstone ileus largely depends on the patient's clinical stability, existing comorbidities, and intraoperative findings. The literature outlines three primary operative strategies: enterolithotomy alone, enterolithotomy combined with simultaneous cholecystectomy and fistula closure (a one-stage procedure), and staged procedures in which definitive biliary surgery is postponed until the patient stabilizes [7,9]. While enterolithotomy alone is the most frequently performed and often adequate procedure, it fails to address the underlying cholecystoenteric fistula, which may predispose patients to recurrent gallstone ileus [6,7]. In our case, the patient required segmental bowel resection due to transmural ischemia and questionable bowel viability at the site of gallstone impaction, making simple enterolithotomy insufficient. This clinical decision is consistent with existing recommendations that advocate segmental resection in cases of bowel necrosis, suspected perforation, or significant stricture formation at the obstruction site [7-9].

The prognosis of gallstone ileus is heavily influenced by the timeliness of diagnosis, prompt surgical intervention, and the presence of comorbid conditions. Although this condition is associated with mortality rates between 12% and 27%, early and appropriate surgical management significantly reduces complications and improves survival outcomes [2,7,9]. Common postoperative complications reported in the literature include anastomotic leakage, wound infection, recurrent obstruction, and persistent biliary fistulas [7,9,10].

## Conclusions

This case underscores the critical importance of considering gallstone ileus in elderly males with no prior surgeries and non-specific symptoms; a high index of suspicion is crucial. The role of early CT scanning in the diagnostic process, particularly in identifying Rigler's triad (pneumobilia, bowel obstruction, and an ectopic gallstone visible on CT), is a key factor that can significantly expedite early operative management.

Drawing from the insights of this case, several essential take-home messages can be identified: implement a two-stage surgical approach, starting with immediate relief of obstruction, followed by staged biliary repair, accompanied by thorough follow-up to avert recurrence. This case report provides practical guidance that supports quicker diagnosis and safer surgical decision-making, thereby minimizing morbidity in similar cases and offering a comprehensive understanding of the intricate considerations involved in managing gallstone ileus with atypical presentations.
